# Systematic Balance Exercises Influence Cortical Activation and Serum BDNF Levels in Older Adults

**DOI:** 10.3390/jcm8111910

**Published:** 2019-11-07

**Authors:** Jadwiga Kubica, Jadwiga Szymura, Aleksandra Domagalik, Slawomir Golda, Magdalena Wiecek, Magdalena Fafrowicz, Tadeusz Marek, Joanna Pera

**Affiliations:** 1 Institute of Physiotherapy, Faculty of Health Science, Jagiellonian University Medical College, 31-126 Krakow, Poland; 2 Department of Clinical Rehabilitation, Faculty of Motor Rehabilitation, University of Physical Education in Krakow, 31-571 Krakow, Poland; jadwiga.szymura@awf.krakow.pl; 3 Brain Imaging Core Facility, Malopolska Centre of Biotechnology, Jagiellonian University, 30-387 Krakow, Poland; aleksandra.domagalik@uj.edu.pl; 4 Department of Molecular Neuropharmacology, Institute of Pharmacology, Polish Academy of Sciences, 31-343 Krakow, Poland; slawomir.golda@gmail.com; 5 Department of Physiology and Biochemistry, Faculty of Physical Education and Sport, University of Physical Education in Krakow, 31-571 Krakow, Poland; magdalena.wiecek@awf.krakow.pl; 6 Department of Cognitive Neuroscience and Neuroergonomics, Institute of Applied Psychology, Jagiellonian University, 30-348 Krakow, Poland; magda.fafrowicz@uj.edu.pl (M.F.); marek@uj.edu.pl (T.M.); 7 Department of Neurology, Faculty of Medicine, Jagiellonian University Medical College, 31-503 Krakow, Poland; joanna.pera@uj.edu.pl

**Keywords:** neuroplasticity, balance training, older adult, fMRI

## Abstract

We sought to investigate whether systematic balance training modulates brain area activity responsible for postural control and influence brain-derived neurotrophic factor (BDNF) mRNA protein expression. Seventy-four older adults were randomly divided into three groups (mean age 65.34 ± 3.79 years, 30 females): Classic balance exercises (CBT), virtual reality balance exercises (VBT), and control (CON). Neuroimaging studies were performed at inclusion and after completion of the training or 12 weeks later (CON). Blood samples were obtained to measure BDNF expression. The study revealed significant interaction of sessions and groups: In the motor imagery (MI) condition for supplementary motor area (SMA) activity (F_at peak_ = 5.25, *p* < 0.05); in the action observation (AO) condition for left and right supramarginal gyrus/posterior insula (left: F_at peak_ = 6.48, *p* < 0.05; right: F_at peak_ = 6.92, *p* < 0.05); in the action observation together with motor imagery (AOMI) condition for the middle occipital gyrus (laterally)/area V5 (left: F_at peak_ = 6.26, *p* < 0.05; right: F_at peak_ = 8.37, *p* < 0.05), and in the cerebellum–inferior semilunar lobule/tonsil (F_at peak_ = 5.47, *p* < 0.05). After the training serum BDNF level has increased in CBT (*p* < 0.001) and in CBT compared to CON (*p* < 0.05). Systematic balance training may reverse the age-related cortical over-activations and appear to be a factor mediating neuroplasticity in older adults.

## 1. Introduction

Postural control involves controlling the body’s position in space for purposes of stability and orientation and plays an important role in daily life. Postural stability is defined as the ability to maintain the projected center of mass (COM) within the limits of the base of support (BOS) [1]. Age-related structural and functional changes of the central nervous system affect brain areas responsible for controlling postural tasks. Changes of postural stability, which are indicated by an increased postural sway and variation of center of foot pressure (COP), inability to execute effective stepping responses, higher risk of falls, and related to injuries morbidity are observed with aging [2]. Postural control also involves cortical and subcortical brain regions apart from brainstem and spinal structures [3]. Sensory and motor areas of the cerebral cortex, the cooperation of which is important in the process of postural control, are activated during the performance of motor tasks [4].

Neuroimaging studies demonstrated the importance of the primary motor cortex (M1), premotor cortex (PMC), supplementary motor area (SMA), and prefrontal cortex (PFC) in the control of postural tasks [5,6]. Involvement of the cortical structures in postural control were also studied in people with damage to the sensory motor and parietal temporal cortex, where greater amplitude of trunk deflection, a decrease in postural response as a reaction to balance disturbances occurring in standing position, were observed [7,8].

Elderly participants, compared to young adults, shift from an automatic to a more attentional, cortical postural control strategy [9]. There is greater cortical activation of M1, PMC, PFC, probably due to compensatory processes that were assumed to counteract the effects of structural damage and the decline of sensory input [6]. The degree of so called “over-activation” is positively correlated with task performance in older adults but it is still debated whether it is related to compensatory processes or dedifferentiation of (i.e., less distinctive) representations [4,10,11].

Physical exercise can induce structural plasticity in the human brain. Exercises modulate activity of the M1, PMC, SMA, and cerebellum, as shown by functional magnetic imaging (fMRI) [12,13]. Balance training may induce changes in the postural control of older adults. Mental simulation of balance tasks has recently been shown to be effective in the activation of brain areas responsible for postural control [6]. fMRI studies using motor imagery (MI), action observation (AO), and the combination of the two (AOMI—Observing while imagining the same action) of balance tasks revealed age-related cortical over-activations and changes in the control of upright posture in comparison to young adults [3]. The results obtained by Ruffieux et al. [9] showed reductions in brain activity during AOMI of the balance task in areas relevant for postural control, including motor, premotor, and multisensory vestibular areas. Applying AOMI of mentally stimulated dynamic balance tasks showed larger brain activations in seniors compared to young participants, while during the MI task (with no visual input), the elderly participants revealed deactivation of subcortical areas [3]. AOMI has repeatedly been demonstrated to be more effective than AO or MI alone [3]. It is known that for postural control, elderly people depend more strongly on visual information than young adults do, thus, we speculated that AOMI might be beneficial in the elderly to activate not only cortical but also subcortical brain areas that are important for postural control due to their stronger dependency on visual input with age. Due to the fact that aging affects postural control, it also influences the way the central nervous system adapts to balance training. It is known that the central nervous system shows plasticity in response to training [14]. Properly selected training can therefore reduce balance disorders and the risk of falls among the elderly group [15]. In addition to the recognised forms of therapy, carried out in the form of gym exercises or at a rehabilitation unit, virtual reality exercises are being increasingly used in the rehabilitation process. The application of biofeedback in exercise-based intervention protocols has previously been proven useful. Due to the extended waiting time, limited availability of classical rehabilitation, and instant feedback on current performance, the use of Nintendo Wii balance training appears to be an important and relevant psychological motivating factor, especially for elderly participants.

In the conducted research, we attempted to find the answer to the question whether balance exercises using the Wii Fit Balance Board can be as effective as other forms of balance therapy [16,17,18]. Studies on virtual reality balance training in older adults revealed improvement in postural control and reaction time. In them, it was indicated that virtual reality exercises may be of use to effectively promote physical fitness in the group of older people [19]. The mechanisms responsible for postural control are subject to neuroplasticity processes, therefore, the use of balance training may not only improve the ability to maintain balance, but also evoke reduction of cortex activation involved in the process of postural control [20].

The brain-derived neurotrophic factor (BDNF), the concentration of which may be regulated by physical exercises, is involved in the neuroplasticity process. The mature BDNF seems to mediate activity-dependent synaptic plasticity [21]. BDNF is a member of the neurotrophin family of growth factors, widely distributed in the central nervous system (CNS) and highly expressed in the mammalian hippocampus and cerebral cortex. It promotes growth and the survival of neurons, regulation of axonal and dendritic branching, and synaptic transmission [22,23,24]. Exercises increase BDNF levels in the brain and serum [25,26,27]. In studies including humans, a change in serum BDNF depending on the intensity and duration of exercises was reported [28,29,30]. From the proposed exercises that have significant impact on changes in BDNF concentration, the most effective seems to be the use of moderate intensity training [30]. In healthy people, the exact mechanism of action of neurotrophins has not yet been fully elucidated and so far, no studies have been conducted on the influence of moderate intensity balance training carried out in two different forms on BDNF protein expression. BDNF concentration decreases significantly with age, therefore, it seems important to study the relationship between the ability to maintain balance and the concentration of this neurotrophin in the peripheral blood of the elderly group participants.

BDNF protein concentration and fMRI brain activation patterns are biomarkers that capture treatment effects and provide information for promoting adaptive neuroplastic changes. To the best of our knowledge, no previous studies have examined the potential efficacy of Nintendo Wii balance training on functional changes in the brain areas responsible for postural stability and BDNF mRNA protein expression in comparison to classical balance exercises. In this study, we sought to investigate whether systematic balance training conducted in a form of classic balance exercises and virtual reality balance exercises modulate brain area activity responsible for postural control analysed with fMRI and influence BDNF mRNA protein expression. In response to mental stimulation of the balance task, we assumed observation of greater activation in cortical and less activation in subcortical areas. We hypothesised that brain activation would be most pronounced during AOMI conditions and less prominent during passive AO or MI alone. We also hypothesised that systematic balance training would decrease activation of brain areas responsible for postural control and elicit more explicit modulation of BDNF protein levels and BDNF mRNA expression in the peripheral blood of older adults. We further hypothesised that there would be a difference in the measured parameters according to the type of moderate intensity balance training.

## 2. Material and Methods

### 2.1. Study Participants

Seventy-four healthy older adults participated in the study (mean age 65.34 ± 3.79 years, 30 females). The inclusion criteria were as follows: Age above 60 years, no medication intake that influences CNS functioning (neuroleptics, antidepressants), no symptoms of cognitive impairment measured by the Mini-Mental State Examination (MMSE ≥ 27), physical fitness that allowed to participate in the test and exercise programme, right-handedness, normal or corrected-to-normal vision, no neurological, orthopaedic or psychiatric disorders. Subjects with MMSE < 27, previous stroke and severe traumatic brain injury, CNS diseases, diabetes or contraindications to fMRI were excluded. All the participants provided their informed consent. All subjects underwent a neurological examination before inclusion. The study was approved by the Jagiellonian University Bioethics Committee and was in accordance with the Declaration of Helsinki.

### 2.2. Experimental Design

Subjects were randomly divided into three groups dependent on experimental intervention: (1) Classical balance training group (CBT) (*n* = 28, 15 females, mean age 66.32 ± 0.60 years), (2) virtual reality balance training group (VBT) (*n* = 15, six females, mean age 65.27 ± 0.85 years), and (3) control group (CON) (*n* = 23, nine females, mean age 64.39 ± 0.99 years).

### 2.3. Somatic Measurements and Body Composition Assessment

Before beginning the balance training, body mass (Jawon IOI-353 Body Composition Analyzer, Gyeongsan, Korea) and body height (Seca 217, Hamburg, Germany) were measured.

### 2.4. Gait and Postural Control Measurement

The Tinetti Performance-Oriented Mobility Assessment (POMA) test was used twice to assess gait and postural control: 1) At inclusion into the study, and 2) after completion of the training cycle or—control subjects—12 weeks following completion [31].

### 2.5. Training

Participants took part in the 12-week training programme under the supervision of a physiotherapist. There were three therapy sessions per week (every other day). In order to gradually adapt participants to the training, in the first week, training sessions lasted 30 min and then 60 min. Training intensity was monitored based on heart rate (Polar heart rate monitor, RSX400, Polar Electro Oy, Kempele, Finland) and was determined at 60%–70% of maximum heart rate which was calculated based on the formula: 208 − 0.7 × age [32]. We indicate that using the HR_max_ equation may not accurately predict an individual’s measured-HR_max_ due to inter-individual variation, although the HR_max_ formula by Tanaka et al. appears to slightly improve the prediction of an individual’s HR_max_ as compared to the HR_max_ formula by Fox et al. [33]. Subjects from the classical training group took part in balance training held in groups of five people. Subjects from the virtual reality training group took part in individual balance training on Wii Fit Balance Board (Nintendo^®^, Redmond, WA, USA). The session was divided into three stages: 1) 5-min warm-up, 2) balance training lasting 50 min (in the first week, 20 min), 3) 5-min cool-down. The CON subjects did not participate in any of the training sessions. For the CBT group, exercises with changes in the support plane were carried out in association with movement around the COM, with minimal use of upper limb stance. Exercises that challenge the center of mass while the feet remain fixed and exercises that practice a narrow base of support (e.g., tandem stance, single-leg stance), were performed. Training included functional balance activities such as gait training, turning, and dual-task training [34]. Dual-task training involved performing a primary task (maintaining postural control on a Swiss exercise Ball or pad) while executing a secondary task (e.g., a manual task such as carrying an item, throwing a ball or a ringo). Dynamic activities were functional and included reaching, stair-stepping, double- and single- leg stance while reaching in any direction, throwing and getting a ball while in standing position. 

Subjects from the VBT took part in individual balance training on the Wii Fit Balance Board. On the qualification day, all subjects played eight games, giving 7 min for each task. The obtained results were used in the selection of specific games for the 12-week training period according to the principle: Selected three task-games, in which the participant received the least number of points on qualification day, plus two others, which were chosen from the remaining five. Participants played each of the five games selected during qualification day for 10 min.

The personal profile of the participant was entered into the system so that the progress could be tracked and the difficulty levels adjusted to each person’s training programme. The system provided an on-screen trainer to lead the user through each exercise and demonstrate proper form. The participants were instructed to step on the Wii Fit balance board, and follow the instructions for the different games available. The list of games used for the balance programme includes: Soccer heading, where the participant had to head soccer balls by moving the head from side to side; ski slalom, where the participant had to ski downhill negotiating gates by shifting body weight from side to side; ski jump, where the participant had to go down a ramp and take-off by bending knees, jump by extending knees, and attempt to land as far down on the hill as possible; table tilt, where the participant had to shift body weight in multiple directions to get balls into a series of holes; obstacle course, where the participant had to navigate through moving platforms, jump to other platforms while avoiding dynamic obstacles; river bubble, where the participant attempted to navigate down a twisting river in a bubble by shifting weight without bursting the bubble on the river banks; perfect 10, where the participant had to shake hips to add up to the given number, desert course, where the participant had to walk to deliver delicious sweets to the hungry guests while balancing a tray. The games were based on the control of an on-screen avatar using body movements that were detected by the balance board.

### 2.6. Functional Magnetic Resonance Imaging

#### 2.6.1. Procedure

Neuroimaging studies were performed twice: 1) At inclusion into the study, and 2) after completion of the training cycle or—control subjects—12 weeks following completion. During each session of fMRI scanning, the subjects performed the experimental task. The task was presented on a 32-inch screen located behind them, from a mirror placed on the head coil. The subjects were informed about the instructions and when ready to start the task, they pressed a button on the Celeritas Fiber Optic Response System (©Psychology Software Tools, Inc. Pittsburgh, PA, USA). The instruction and task commands were presented both on the screen and through auditory command. The auditory system of the NordicNeuroLab (NordicNeuroLab AS, Bergen, Norway) was used. The whole fMRI session lasted about 25 min.

#### 2.6.2. Experimental Task

**Figure 1:**
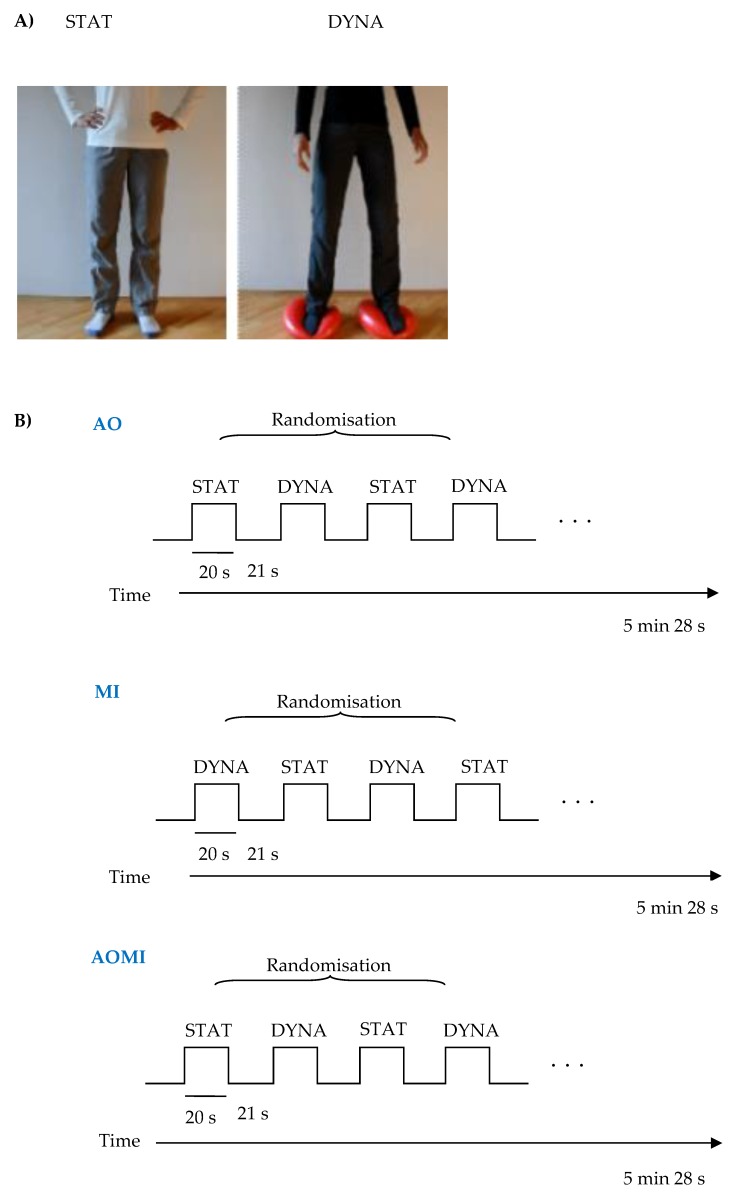
Experimental design. (**A**) Display of the static and dynamic balance task, respectively. (**B**) Scheme of the study performed with functional magnetic resonance imaging (fMRI). AO—action observation, MI—motor imagery, AOMI—motor imagery with action observation, STAT—standing position without moving, DYNA—participant had to balance on two balance pads.

A modified version of the task proposed by Taube et al. [35] was used. The task introduces three conditions: Motor imagery (MI), action observation (AO), and action observation together with motor imagery (AOMI). Before each condition, the instruction was presented on the screen and via headphones. In every condition, two types of stimuli were presented for 20 s: Dynamic standing balance (DYNA; where participant had to balance on two balance pads (Dynair® senso 36 cm TOGU®, GmbH, Prien am Chiemsee, Germany), shifting the body weight from one side to the other) or static standing balance (STAT; a non-moving character in a standing position). These stimuli were presented four times each in a random order. After each stimulus, there was a 21 s rest period with a black cross displayed on the center of the screen. In total, each condition lasted 5 min and 28 s. During MI, participants were asked to imagine themselves performing the presented stimuli. At the beginning of the stimuli, auditory and visual cues were presented, providing information about the type of stimuli (e.g., the word “balance” appeared on the screen and the auditory command was presented via the headphones). Similarly, at the end of the stimuli, the auditory and visual cues were presented informing about the end of action. Participants were asked to close their eyes during this kinesthetic imagery. During AO and AOMI conditions, subjects were presented with a 20 s video showing each type of stimuli. The person on the video wore different-coloured clothes to differentiate the condition. In the AOMI condition, subjects were instructed to watch the video of a person performing the balance task and, at the same time, imagine performing the task themselves. Subjects were asked to imagine themselves as the person on the video shown in a mirror. It has been proven that mirror images facilitate imitation and observational learning [35]. In AO conditions, they were instructed just to watch the video and not refer it to themselves (Figure 1). Before entering the fMRI scanner session, subjects were instructed on how to perform the task and viewed the short version of the task in front of the computer screen. They also practiced the dynamic standing balance task.

#### 2.6.3. Magnetic Resonance Imaging Data Acquisition and Analysis

MRI was performed using a 3T scanner (Magnetom Skyra, Siemens, Healthcare Diagnostics, Inc., Tarrytown, NY, USA) with a 20-channel head/neck coil. High-resolution, anatomical images were acquired using a T1 MPRAGE sequence (sagittal slices; 1×1×1.1 mm^3^ voxel size; TR = 2300 ms, TE = 2.98 ms). Next, a B0 inhomogeneity gradient field map (magnitude and phase images) was acquired with a dual echo gradient echo sequence matched spatially with fMRI scans (TE1 = 4.92 ms, TE2 = 7.38 ms, TR = 508 ms). Spatial parameters were adjusted to match the EPI sequence. Functional images were acquired using an EPI sequence. The scan parameters were as follows: mm isotropic voxel, TR = 2700 ms, TE = 30 ms, flip angle = 90°, FOV 192×192 mm^2^, GRAPPA acceleration factor 2, phase encoding A/P. The whole brain image (cerebellum included) was covered with 48 axial slices, taken in an ascending manner. The acquisition time for each run was 5 min and 30 s. There were three scanning runs, one for each task condition. Due to magnetic saturation effects, the first four volumes (dummy scans) of each run were acquired and then discarded by the scanner.

fMRI analysis was carried with the Analysis of Functional NeuroImage (AFNI) software (version 18.1.33; National Institute of Mental Health, Rockville, MD, USA) [36,37] Anatomical images were skull-stripped and normalised into the coordinate system of Montreal Neurological Institute (MNI) space. Functional data pre-processing procedure included despiking, slice timing correction, head motion correction. Then, the FUGUE toolbox, part of FSL (FMRIB’s Software Library, www.fmrib.ox.ac.uk/fsl; Wellcome Centre for Integrative Neuroimaging FMRIB, John Radcliffe Hospital Headington, Oxford, UK), was used to correct for B0 inhomogeneity using a gradient field map. Next, functional data were co-registered to structural scans, the signal was scaled to represent percentage changes and then data were spatially smoothed using full-width at a half maximum isotropic Gaussian kernel of 6 mm. Finally, data were normalised into the coordinate system of MNI space.

The subject-level statistical analysis was performed using generalised least squares implemented in the 3dREMLfit script in AFNI that uses the auto-regressive moving-average (ARMA) model. Each trial, i.e., dynamic stimulus (DYNA) and static (STAT), were modelled as 20 s blocks for each task. Additionally, for the MI task, the occurrence of the command at the end of block (auditory stimulus to stop performing the task) was convolved with the canonical double-gamma model of the hemodynamic response function (HRF) and its temporal derivative. Finally, 12 movement parameters (six original and its first derivatives) were included in the model as nuisance regressors as well as higher-order polynomial accounting for slow drifts in the fMRI time series. At this level, the active condition contrast was defined, i.e., DYNA versus STAT.

#### 2.6.4. Statistical Analysis

During the fMRI group analysis, the T-test was performed on the data from all subjects at baseline sessions, for each condition separately. This step created general maps of activation for MI, AO, and AOMI tasks. The maps were thresholded at *p* < 0.001 and corrected for multiple comparisons with the false discovery rate method and at a minimum cluster size of 15 voxels. Next, the ANOVA test was performed on the data from all subjects using the 3dMVM script in order to check the interaction effect of the ‘group’ (three levels: Classical, virtual, and control group) and ‘sessions’ (two levels accounting for baseline and the session after the experimental intervention). The general maps of activation for each condition described above were used as masks during this analysis. The beta values for each subject, each session, and each condition were extracted from significant clusters. The post-hoc test was calculated with the Statistica software (StatSoft, Inc., Tulsa, OK, USA). Data distribution was assessed by the Shapiro–Wilk test. The significance of intergroup differences for single measurements, depending on the distribution of variables, was estimated with the variance analysis test. Analysis of variance with repeated measurements (ANOVA) for each dependent variable was used to assess the impact of the 12-weeks of balance training among the compared groups (CBT, VBT, CON). ANOVA analysis allowed to evaluate the impact of the main factors on the measured variables, i.e., group (form of balance training used (CBT, VBT) or lack of training (CON)), training (repeated measurement—before training, after training) and the interaction of these factors group×training. In the case of significant influence of any of the main factors, i.e., group, training, and group×training interactions, the significance of differences between specific averages was confirmed by performing statistical post-hoc analysis (Tukey’s HSD test for different n). The statistical significance of differences between the compared averages was assumed at the level of *p* < 0.05. The STATISTICA 13.0 package was used.

### 2.7. Blood Sampling

Blood samples were obtained by venipuncture from the antecubital vein in the morning hours (8:00–10:00 am) using the BD Vacutainer®vacuum system (Becton Dickinson, Franklin Lakes, NJ, USA) in a seated position prior to the first balance training and after 12 weeks of exercises. In the controls, blood samples were obtained at the beginning of the experiment and after 12 weeks. For the assay of BDNF serum, test tubes with a clotting activator were used; after the collection of blood, these were stored for 20 min at 20–22 °C until a clot was obtained, and then they were centrifuged (relative centrifugal force 1.000 g) for 15 min at 4 °C using MPW-351R centrifuge (MPW Med. Instruments, Warsaw, Poland) and the supernatant was decanted and stored at −75 °C (ULUF 450 Arctiko, Esbjerg, DK) until analysis.

#### 2.7.1. Serum BDNF Measurements

BDNF serum concentration was assayed using the DBNT00 (R&D Systems Inc., Minneapolis, MN, USA) according to the manufacturer’s instructions. Determinations were performed using the immunoenzymatic method (ELISA) with the measurement of absorbance via the E-LizaMat 3000 microplate reader (DRG International, Inc., Springfield, NJ, USA) according to the methodology presented by the manufacturer, reading the results from the standard curve performed during each test (R&D Systems, Minneapolis, MN, USA) with reference to the manufacturer’s instructions. The sensitivity of BDNF for this test was 0.997 pg/mL, the inter- and intra-assay were < 7.2% and < 3.2%, respectively.

#### 2.7.2. BNDF mRNA Expression Measurements

Venous whole blood was collected in PAXgene Blood RNA Tubes (Qiagen, PreAnalytiX, Hombrechtikon, Switzerland) and was mixed and kept at room temperature for at least 2 h. The tubes were track frozen and stored at –20 °C until further processing. Total RNA was purified from blood samples using the PAXgene Blood RNA Kit (Qiagen) according to the manufacturer’s protocol and was treated with DNase. RNA concentrations were measured using a NanoDrop ND-1000 Spectrophotometer (NanoDrop Technologies, Montchanin, DE, USA), and RNA quality was determined by chip-based capillary electrophoresis utilising an RNA 6000 Nano LabChip Kit and an Agilent Bioanalyzer 2100 (Agilent, Palo Alto, CA, USA) according to the manufacturer’s protocols. Reverse transcription (RT) was performed using an Omniscript RT Kit (Qiagen, Valencia, CA, USA) at 37 °C for 60 min. RT reactions were performed in the presence of an RNase inhibitor (rRNasin; Promega, Madison, WI, USA) and an oligo (dT12–18) primer (Invitrogen, Life Technologies, Carlsbad, CA, USA). Next, cDNA was diluted 1:10 with H_2_O, and approximately 40 ng of cDNA synthesised from the total RNA template extracted from the individual’s blood individual was used for each reaction. The qPCR reactions were performed utilising TaqMan probes for human *HPRT1* (Hypoxanthine Phosphoribosyltransferase 1) (probe id: Hs02800695_m1) and *BDNF* (probe id: Hs02718934_s1) transcripts in accordance with the manufacturer’s protocol (Applied Biosystems, Foster, CA, USA), and were run on a CFX96 Real-Time System with a C1000 Touch Thermal Cycler (BioRad, Hercules, CA, USA) using Bio-Rad CFX Manager 2.1 software. The cycle threshold values were automatically calculated with default parameters. The transcript abundance of *BDNF* was normalized to *HPRT1* level and calculated as 2^−(dCt) by Bio-Rad CFX Manager 2.1 software (where dCt denotes: Threshold cycle for BDNF— threshold cycle for HPRT1).

## 3. Results

Three subjects were excluded from the analysis because they interrupted the experiment. Five more subjects were excluded due to movement artefacts in the MR scanner. No statistical differences were found between groups before the study. Baseline characteristics of the groups are shown in Table 1.

### 3.1. Postural Control Results

**Figure 2:**
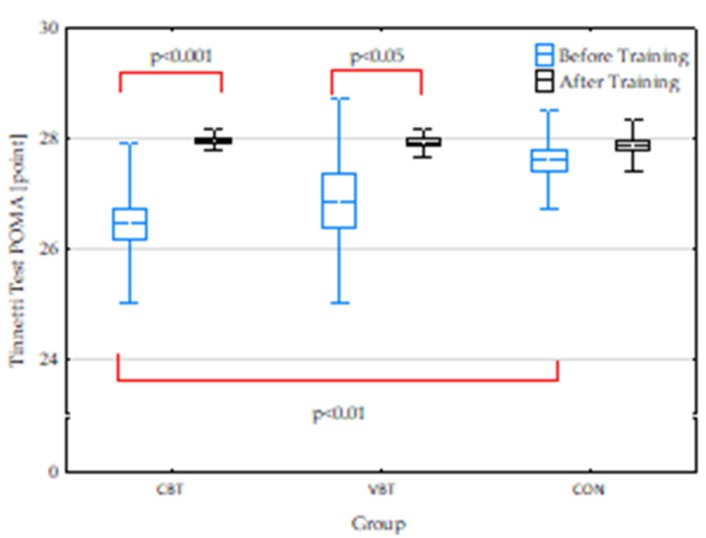
Tinetti (POMA) results before and after completion of 12-week balance training. Presentation of individual data, mean (---), SEM (box), and SD (whiskers); CBT—classical balance training, VBT—virtual reality balance training, CON—control group; significant differences: *p* < 0.05

At baseline, we found statistical differences in the Tinetti (POMA) results only for the CBT and CON groups. Subjects from the CBT group had significantly lower results than subjects from the CON (*p* = 0.002). Twelve weeks of systematic balance training induced significant changes in Tinetti (POMA) results in subjects from CBT and VBT (*p* = 0.000, *p* = 0.034). Tinetti (POMA) results for the CON group were similar at baseline and after 12 weeks (Figure 2).

### 3.2. Functional Magnetic Resonance Imaging Results

**Figure 3:**
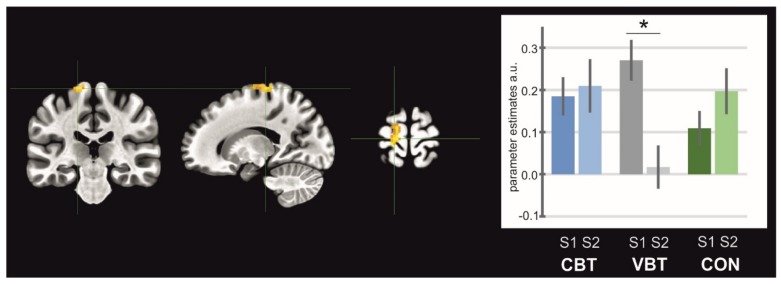
Brain structure (supplementary motor area) showing interaction between groups and sessions for MI task. Note: CBT—classical balance training, VBT—virtual reality balance training, CON—control group; asterisk indicates significant differences between sessions.

**Figure 4:**
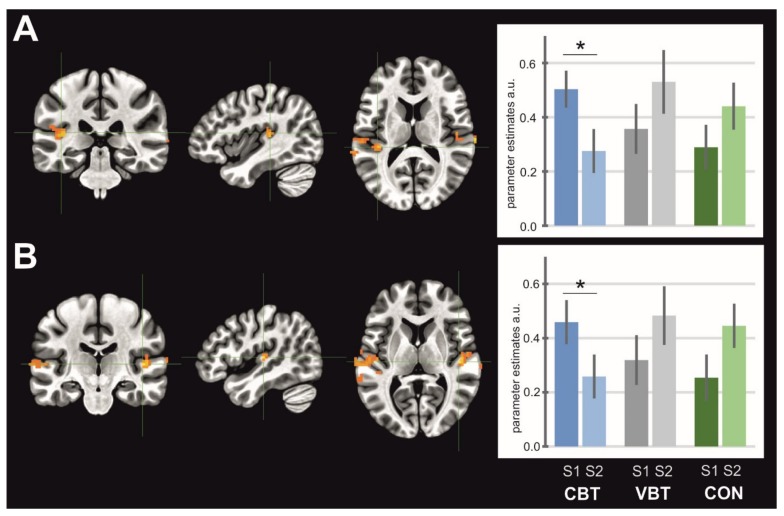
Brain structures showing interaction between groups and sessions for AO task; (**A**) left supramarginal gyrus/posterior insula, (**B**) right supramarginal gyrus/posterior insula. Note: CBT— classical balance training, VBT—virtual reality balance training, CON—control group; asterisk indicates significant differences between sessions.

**Figure 5:**
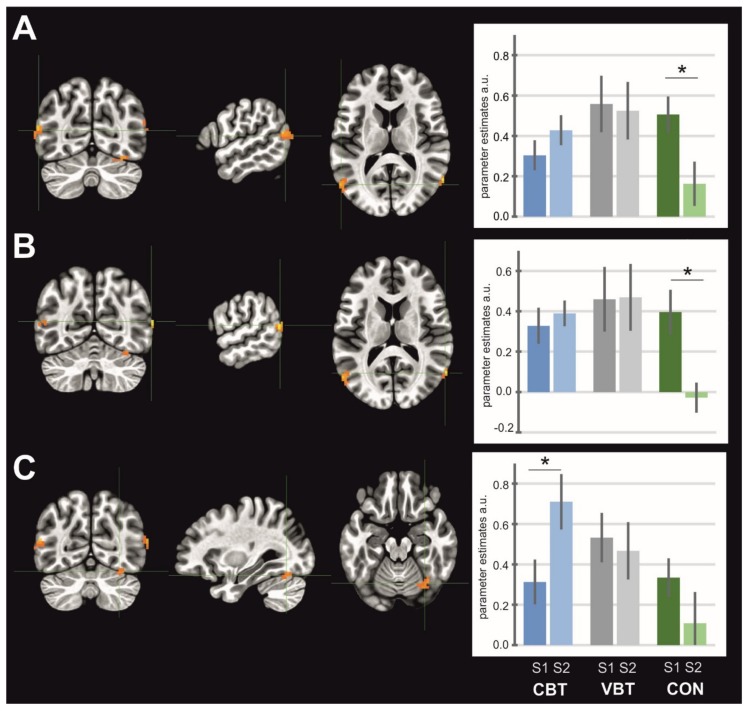
Brain structures showing interaction between groups and sessions for AOMI task; (**A**) left middle occipital gyrus (laterally)/area V5, (**B**) right middle occipital gyrus (laterally)/area V5, (**C**) cerebellum–inferior semilunar lobule/tonsil. Note: CBT—classical balance training, VBT—virtual reality balance training, CON—control group; asterisk indicates significant differences between sessions.

General maps of activation for MI, AO, and AOMI revealed activity in a wide set of brain areas. All significantly activated clusters are listed in Table 2, Table 3, and Table 4 for each condition, respectively. The ANOVA test revealed significant interactions of sessions and groups for SMA activity in the MI condition (F_at peak_ = 5.25, *p* < 0.05, Figure 3), where the post-hoc test indicated a significant decrease in the activity of this structure for VBT (*p* = 0.03). In the AO condition, there was significant interaction for the left and right supramarginal gyrus/posterior insula (left: F_at peak_ = 6.48, *p* < 0.05, Figure 4A; right: F_at peak_ = 6.92, *p* < 0.05, Figure 4B). Both clusters showed decrease in activity for the CBT group (left: *p* = 0.01; right: *p* = 0.04). In the AOMI condition, interactions of groups and sessions were found bilaterally in the middle occipital gyrus (laterally)/area V5 (left: F_at peak_ = 6.26, *p* < 0.05, Figure 5A; right: F_at peak_ = 8.37, *p* < 0.05, Figure 5B), and in the cerebellum–inferior semilunar lobule/tonsil (F_at peak_ = 5.47, *p* < 0.05, Figure 5C). Activity in the middle occipital gyrus (laterally)/area V5 significantly decreased for the CON (left: *p* = 0.0004; right: *p* = 0.02), whereas in the cerebellum—inferior semilunar lobule/tonsil activity increased for the CBT (*p* = 0.01).

### 3.3. Serum BDNF Expression

**Figure 6:**
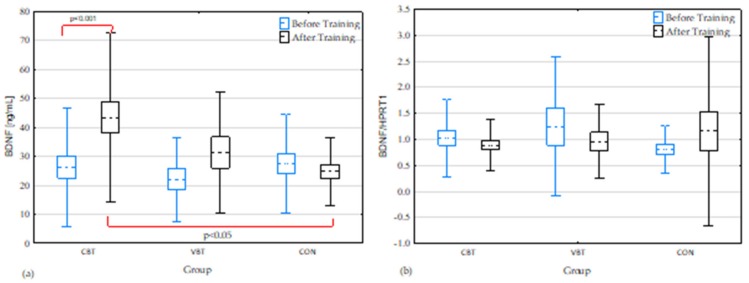
BDNF concentrations (**a**) and expression (**b**) in the blood before and after completion of the 12-week balance training. Significant differences: *p* < 0.05; mean (---), SEM (box), and SD (whiskers); CBT—classical balance training, VBT—virtual reality balance training, CON—control group; BDNF—brain-derived neurotrophic factor, *HPRT1*—Hypoxanthine Phosphoribosyltransferase 1.

The baseline serum BDNF protein level did not differ between groups. After completion of 12-weeks of moderate-intensity balance training, BDNF serum levels increased significantly in the CBT group compared to baseline levels (*p* < 0.001). There was a significant difference in BDNF serum level between the CBT and CON groups after 12 weeks (*p* < 0.05) (Figure 6a).

BDNF mRNA levels did not differ significantly before or after completion of the 12-week balance training or between groups (Figure 6b).

## 4. Discussion

In the present study, we investigated effects of 12-week balance training of moderate intensity conducted in two different forms on brain activity during motor simulation of a balance task and BDNF expression in the blood of older adults.

After completion of the 12-week training programme, we observed improvement in the Tinetti mobility test results in both training groups. No differences were found in the CON group. Further, we found that systematic balance training caused a significant reduction in the activity of cortical areas responsible for postural control. Adaptations of BNDF level were observed only in the CBT group.

To the best of our knowledge, the present study is the first to examine the effect of two different types of 12 weeks of moderate intensity balance training, namely, classical and in virtual reality, on brain activation patterns, BDNF protein level and BDNF mRNA expression in the peripheral blood of older adults.

### 4.1. General Maps of Activation of Brain Areas in MI, AO, AOMI Conditions

Activation in MI revealed activity of the SMA, supramarginal gyrus (SMG) middle frontal gyrus and cerebellum. Lack of M1 activation is in line with the results of previous studies, where in the absence of sensory information, areas associated with imagined movement were activated although the actual movement was not performed [38]. It is known that SMA may inhibit M1 activity during MI conditions, indicating the occurrence of a closed loop of control between SMA and M1 [39]. Inhibition of M1 may also increase with aging [40]. The cerebellum and parietal lobe are involved in predicting the sensory consequences of movement, and together with the premotor cortex, they are activated by both imagining and executing movement. Since the parietal cortex receives information from the cerebellum via the thalamus and has a connection in the opposite direction via the pons, it is likely that these two regions work in parallel to predict the sensory consequences of movement by monitoring and making corrections according to the movement [41]. Older people shift from an automatic to a more cortical postural control strategy [4,41], and in comparison to the younger cohort, they show larger cortical activations and deactivation of subcortical regions, especially during a challenging dynamic task [3,9]. Comparison of the MI and AO conditions in fMRI tests revealed that older participants were characterised by reduced activation of subcortical structures and the cerebellum in the absence of visual stimuli (MI), indicating that older persons rely more on visual support during the performance of more automated postural tasks [3].

In AO conditions, we found activation in the middle occipital gyrus (laterally)/area V5, SMG, SPL, superior and middle frontal gyrus (SFG, MFG), and precuneus.

Over-activation of cortical areas responsible for movement perception and positioning of the body in space (MT/V5 - middle temporal area) and the somatosensory cortex, is accompanied by a reduction in subcortical activation in elderly people. This may be caused by reduced reciprocal inhibitory sensory interaction, which as a compensation mechanism, affects more conscious control of posture [42].

In the study by Eaves et al. [43], the authors suggested that AOMI may enable subjects to acquire better physiological sensations and kinesthetic experiences of the imagined movement [43]. It was proposed that AOMI may be a very promising approach in activating internal movement representations of postural tasks in the elderly [35].

In our study, general maps of activation revealed that AOMI, MI, and AO tasks influenced areas of the brain that are important for postural control. The most pronounced brain activation was observed in AOMI conditions. This is in line with previous studies in which brain activations in AOMI were not simply the addition of activity of independent AO and independent MI, but were significantly larger than the sum of those two conditions [35].

### 4.2. Activation of Brain Areas in Response to Balance Training

In our study, the results revealed a significant interaction of the sessions and groups for: 1) SMA activity in the MI condition, where a significant decrease in activity for the VBT group was observed, 2) left and right SMG/posterior insula in the AO condition, where both clusters showed decrease activity for the CBT group, 3) the middle occipital gyrus (laterally)/area V5 in the AOMI condition, where activity significantly decreased for the CON group, and 4) cerebellum–inferior semilunar lobule/tonsil in the AOMI condition, where a significant increase of activity was observed for the CBT group.

It was originally assumed that the spinal cord and brainstem were involved in postural control process [44]. In later studies, it was shown that cortical and subcortical areas of the brain are also involved in balance activities. Sensory and motor areas of the cerebral cortex, important for postural control, are activated during the performance of motor tasks [4]. It was proved that SMA or M1 areas of the motor cortex are involved in the process of postural control [44]. This especially applies to older people who are believed to change from automated (subcortical) to a more conscious (cortical) way of postural control [45]. In recent studies, it has been indicated that in older adults, overactivation of cerebral cortex areas may occur during the performance of balance activities as a result of functional compensation caused by the progressive disease process of the nervous system or age-related damage of its structure and function. Overactivation of cortical structures is usually accompanied by a decrease in activation of subcortical structures [3,6,40]. The increased activation of specific areas of the cortex, responsible for the execution of motor and cognitive tasks in the elderly, has not been fully elucidated, but the possible explanation is: Compensatory processes or less characteristic diversity of cortical representation [2]. In the elderly, activation of the subcortical areas responsible for balance control in a standing position depend on the occurrence of visual stimuli. Comparison of the MI and AO tasks revealed that older people were characterised by reduced activation of the subcortical structures and the cerebellum in the absence of visual stimuli (MI), indicating that older people rely more on support from the visual system during more automated postural tasks [3]. Characteristic for the elderly, specific overactivation in the area of the cortex responsible for the perception of movement and body position in space (MT/V5) and the sensory-motor cortex (postcentral gyrus), may be caused by reciprocal inhibitory sensory interaction in this area, which as a compensatory mechanism, affects more conscious posture control [42].

Rogge et al. [46] observed that 12 weeks of balance training elicit neuroplasticity in brain regions associated with visual and vestibular self-motion perception. Studies using functional near-infrared spectroscopy suggest that sensorimotor cortical areas are crucial for balance control, pointing out SMA involvement in the control of sway in a mediolateral direction [47]. Reductions in motor, premotor, and multisensory vestibular areas were observed in response to five weeks of balance training during AOMI of the balance task suggesting that balance training may reverse age-related cortical over-activations [9]. Our data also showed a significant training-induced reduction in brain area activity for which over-activations have been previously reported. Completion of the 12-week virtual balance training programme resulted in a significant decrease of SMA activity for the VBT group in MI condition.

In MI condition, brain areas related to the imagined movement are activated, although the actual movement is not performed [38]. Kasess et al. [39] observed that SMA can inhibit M1 activity during an MI task to prevent the actual performance of imagined movement. This may indicate a closed control loop between SMA and M1. Similar relationships were noted in the presented study. In the MI task, for VBT, significant activity was found in SMA, but a lack was noted for M1 and basal ganglia activity, as well as a lack of activity in the area of the visual cortex. In our study, after completion of 12 weeks of moderate intensity VBT, reduction in SMA activity was noted. SMA is activated during learning new motor skills and is responsible for planning and movement control. Together with PFC, it affects postural changes. Thus, the observed reduction in SMA activity may mean that automatism and motor learning was influenced due to the biofeedback used in VBT.

We found reduced activation of the SMG/posterior insula for the CBT group in the AO conditions. Research revealed that during the performance of a task requiring visual attention and motion perception, the left part of the SMG is activated [48]. SMG is a part of the mirror neuron system (MNS) [49]. Activation of MNS neurons in humans occurs during movement and observation of movement performed by someone else as an element of motor learning and converting observations into a new movement skill [50]. Our results indicate that classical balance exercises influenced the automatism and learning of postural activities observed during training, therefore, leading to a reduction of activity in the SMG.

In previous studies, stronger cortical activity was mainly found for the conditions with visual input (AOMI and AO). In the condition without visual input (MI), elderly participants displayed reduced activity in subcortical areas such as the putamen and the cerebellum, indicating that the activation of subcortical representations responsible for the postural control strongly depend on visual input. Activation of visual areas was considered important for automated postural task execution [3]. In our study, a significant increase in the activity of the cerebellum in AOMI condition after completion of CBT showed that classical balance exercises may modulate the postural control strategy by changing from a cortical (conscious) to a more automatic strategy as in younger adults.

The decreased activation of the middle occipital gyrus (laterally)/area V5 in the AOMI condition presented in our study, may indicate occurrence of the priming phenomenon for the CON group. This phenomenon is explained by the fact that earlier stimulus exposure paves the way for potential information processing with the participation of this stimulus and, in a way, anticipates the neuronal system, which requires less recruitment when such information processing occurs.

### 4.3. BDNF Expression

The BDNF protein in the serum concentration increased significantly only in the CBT group after completion of the 12-week balance training in comparison to the CON group and to baseline. We did not find a significant effect of classical or virtual reality balance training on BDNF mRNA expression when compared to baseline.

To the best of our knowledge, this is the first study examining the influence of moderate intensity balance training on the BDNF protein or mRNA expression.

In many studies, the influence of different forms of training with various intensities on BDNF concentration in the blood serum, blood platelets or blood plasma, has been analysed [29,51,52]. The intensity of physical exercise is an important factor affecting the concentration of BDNF. In the vast majority of exercise intervention studies of peripheral BDNF, aerobic training in adults has been examined [51]. In several studies, it has been demonstrated that systemic BDNF levels increase following moderate resistance training [53,54], while others reported no influence [55,56,57]. Analysis of BDNF concentration changes under the influence of high-intensity exercise showed a transient, moderate (approximately 20%–40%) increase in BDNF concentration in the peripheral blood [28,58,59].

We found that only systematic classical balance exercises of moderate intensity significantly increased serum BDNF. The possible explanation for the difference between training groups may be the different nature of virtual reality exercises. Exercises in VBT involved muscle groups using more isometric exercises. CBT involved more concentric and eccentric muscle work. The available literature on the changes in BDNF concentration under the influence of different types of training is essentially associated with its intensity. Our results may indicate that there is a connection between the type of muscle contraction and BDNF concentration. It is probable that the increase of circulating BDNF concentration during exercise in humans may originate from the contracting muscle cells, although further research is required.

Aging or neurodegenerative diseases are associated with a decrease in BDNF expression. BDNF can mediate the neuroprotective effect of physical activity, both in the physiological process of aging and in neurodegenerative diseases [60]. However, in our study, it was revealed that the mRNA level remained unaltered. It has been demonstrated that the main sources of BDNF mRNA are megakaryocytes, the progenitors for platelets. The expression of BDNF, similarly to neurons, strongly depends on calcium levels [61]. One could speculate that long-term classical balance training significantly increased only the protein level of serum BDNF, secreted from platelets, because the calcium level remained stable. Nevertheless, the calcium level in the serum was not assessed, thus, further research is required.

Our study revealed that classical balance training significantly increases the level of serum BDNF. Due to BDNF concentration changes, we suggest that systematic classical balance training of moderate intensity may be a factor mediating neuroplasticity in older adults. 

### 4.4. Limitations of the Study

The findings of this study have to be seen in light of some limitations. A certain limitation was the difference in sample size of the studied groups. The VBT group was relatively small. Further investigation with a larger sample is required to confirm whether this type of training is superior to classical balance training.

## 5. Conclusions

The study revealed that both forms of moderate intensity balance training can affect functioning of the CNS by activation of the sensory and motor cortex involved in postural control. Activation of the sensory cortex associated with the perception of movement in AO and AOMI conditions indicates the involvement of the visual system in performing automated activities by older people. The study confirmed that with age, postural control may become more consciously controlled (cortically). Systematic CBT of moderate intensity may reverse age-related cortical over-activations and appear to be a factor mediating neuroplasticity in older adults due to the modulating effect it also produces on BDNF protein concentration. VBT can complement CBT due to its wide availability and instant feedback on current performance, which may be an important and relevant psychological motivating factor, especially for older people. 

## Figures and Tables

**Table 1:** Baseline characteristics of the groups.

		Group	
Variables	CBT	VBT	CON
	*n* = 28	*n* = 15	*n* = 23
MMSE (points)	29.18 ± 0.18	29.29 ± 0.30	29.43 ± 0.19
Age (years)	66.32 ± 0.60	65.27 ± 0.85	64.39 ± 0.99
Sex (Male/Female)	13/15	9/6	14/9
BH (cm)	166.12 ± 1.65	161.58 ± 7.21	168.20 ± 1.83
BM (kg)	78.09 ± 2.21	83.54 ± 5.61	77.94 ± 3.00
LBM (kg)	53.80 ± 1.98	53.83 ± 3.30	54.64 ± 2.23
PBF (%)	34.74 ± 1.25	33.01 ± 2.99	29.67 ± 1.38
FM (kg)	24.68 ± 1.21	25.12 ± 1.60	23.30 ± 1.37
BMI (kg·m^-2^)	28.31 ± 0.74	28.87 ± 1.15	27.40 ± 0.77

Data are presented as mean ± SD; BH: Body height; BM: Body mass; BMI: Body mass index; CON: Control group; CBT: Classical balance training group; FM: Fat mass; LBM: Lean body mass; MMSE: Mini-Mental state examination; PBF: Percentage of body fat; VBT: Virtual reality balance training group.

**Table 2:** Structures activated during the active contrast task in motor imagery (MI) conditions.

Brain Area	Side	X	y	Z	T	Cluster Size
Supplementary motor area	Medial	−1.1	−13.2	+68.7	8.03	415
Supramarginal gyrus	Left	−56.6	−39.4	+39.4	5.32	48
Middle frontal gyrus	Left	−48.5	−5.6	+56.7	5.61	23
Cerebellum—culmen	right	+28.2	−42.5	−27.6	6.39	31

Note: Coordinates (XYZ) of cluster center-of-mass in the standard MNI (Montreal Neurological Institute) space. Cluster size in the number of voxels.

**Table 3:** Structures activated during the active contrast task in action observation (AO) conditions.

Brain Area	Side	x	y	Z	T	Cluster Size
Middle occipital gyrus (laterally)/area V5	left	−48.3	−72.7	+5.3	11.73	357
	right	+50.0	−65.1	+4.4	12.20	521
Middle occipital gyrus (medially)	left	−14.3	−85.0	−5.7	6.28	102
	right	+16.7	−91.6	+7.1	5.62	52
Supramarginal gyrus	left	−56.8	−31.1	+18.7	8.29	842
	right	+59.6	−27.4	+17.0	8.01	946
Superior parietal lobule	left	−28.7	−52.1	+62.6	5.96	275
	right	+27.9	−49.2	+60.8	6.42	288
Superior frontal gyrus	right	+19.7	−11.0	+67.1	6.31	88
Middle frontal gyrus	right	+46.0	−1.8	+54.3	4.98	51
Middle cingulate cortex	left	−12.7	−26.4	+45.7	6.07	52
	right	+13.4	−29.0	+47.4	5.90	41
Precuneus	right	+24.1	−84.6	+38.2	4.79	65

Note: Coordinates (XYZ) of cluster center-of-mass in the standard MNI (Montreal Neurological Institute) space. Cluster size in the number of voxels.

**Table 4:** Structures activated during the active contrast task in action observation together with motor imagery (AOMI) conditions.

Brain Area	Side	x	y	Z	T	Cluster Size
Supplementary motor area	medial	−0.4	−15.7	+62.8	6.30	828
Middle frontal gyrus	left	−43.9	−6.3	+54.8	6.26	146
	right	+45.2	−1.8	+52.1	5.02	147
Supramarginal gyrus	left	-58.7	−30.6	+20.5	6.53	722
	right	+62.0	−26.9	+16.6	7.58	813
Superior parietal lobule	left	−27.6	−53.3	+63.6	5.72	371
	right	+25.2	−52.3	+62.0	5.05	359
Middle occipital gyrus (laterally)/area V5	left	−47.6	−74.6	+3.9	11.72	442
	right	+50.8	−67.9	+2.6	14.22	506
Middle occipital gyrus (medially)	left	−16.4	−84.4	+2.3	4.99	312
	right	+20.5	−80.3	+3.1	3.71	345
Cerebellum–inferior semilunar lobule/tonsil	left	−25.5	−62.8	−57.1	4.50	23
	right	+26.1	−60.9	−53.7	3.71	28
Superior colliculus	left and right	+1.2	−30.1	−3.3	3.73	25

Note: Coordinates (XYZ) of cluster center-of-mass in the standard MNI (Montreal Neurological Institute) space. Cluster size in the number of voxels.
